# Distinct DNA Exit and Packaging Portals in the Virus *Acanthamoeba polyphaga mimivirus*

**DOI:** 10.1371/journal.pbio.0060114

**Published:** 2008-05-13

**Authors:** Nathan Zauberman, Yael Mutsafi, Daniel Ben Halevy, Eyal Shimoni, Eugenia Klein, Chuan Xiao, Siyang Sun, Abraham Minsky

**Affiliations:** 1 Department of Organic Chemistry, The Weizmann Institute of Science, Rehovot, Israel; 2 Electron Microscopy Center, The Weizmann Institute of Science, Rehovot, Israel; 3 Department of Biological Sciences, Purdue University, West Lafayette, Indiana, United States of America; University of Wisconsin, Madison, United States of America

## Abstract

Icosahedral double-stranded DNA viruses use a single portal for genome delivery and packaging. The extensive structural similarity revealed by such portals in diverse viruses, as well as their invariable positioning at a unique icosahedral vertex, led to the consensus that a particular, highly conserved vertex-portal architecture is essential for viral DNA translocations. Here we present an exception to this paradigm by demonstrating that genome delivery and packaging in the virus *Acanthamoeba polyphaga mimivirus* occur through two distinct portals. By using high-resolution techniques, including electron tomography and cryo-scanning electron microscopy, we show that Mimivirus genome delivery entails a large-scale conformational change of the capsid, whereby five icosahedral faces open up. This opening, which occurs at a unique vertex of the capsid that we coined the “stargate”, allows for the formation of a massive membrane conduit through which the viral DNA is released. A transient aperture centered at an icosahedral face distal to the DNA delivery site acts as a non-vertex DNA packaging portal. In conjunction with comparative genomic studies, our observations imply a viral packaging pathway akin to bacterial DNA segregation, which might be shared by diverse internal membrane–containing viruses.

## Author Summary

Two fundamental events in viral life cycles are the delivery of viral genomes into host cells and the packaging of these genomes into viral protein capsids. In bacteriophages and herpesviruses, these processes occur linearly along the genome, base pair after base pair, through a single portal located at a unique site in the viral capsid. We have addressed the question of whether such a linear translocation through a single portal also takes place for viruses harboring very large genomes, by studying genome delivery and packaging in the amoeba-infecting virus *Acanthamoeba polyphaga mimivirus*. With 1.2 million base pairs, this double-stranded DNA genome is the largest documented viral genome. By using electron tomography and cryo-scanning electron microscopy, we identified a large tunnel in the Mimivirus capsid that is formed shortly after infection, following a large-scale opening of the capsid. The tunnel allows the whole viral genome to exit in a rapid, one-step process. DNA encapsidation is mediated by a transient aperture in the capsid that, we suggest, may promote concomitant entry of multiple segments of the viral DNA molecule. These unprecedented modes of viral genome translocation imply that Mimivirus—and potentially other large viruses—evolved mechanisms that allow them to cope effectively with the exit and entry of particularly large genomes.

## Abstract 3

The mechanisms that promote genome delivery and packaging in the giant virus Mimivirus call into question the conventional distinction between viruses and single-celled organisms.

## Introduction

The prevailing model for genome translocations in icosahedral viruses entails a molecular motor that is localized at a single vertex and comprises a packaging ATPase and a portal complex [1–5]. The particular structural features revealed by the vertex-portal assembly have been argued to facilitate both genome delivery [6] and genome encapsidation [3,6,7]. Although the functional implications of these features have been recently challenged [8,9], their apparent conservation led to the paradigm that a single vertex-portal system plays a crucial and general role in both genome injection and packaging in icosahedral viruses [6].

Vertex-portal assemblies were, however, characterized only in herpesviruses that contain an external lipid membrane [3,4,10], and in tailed double-stranded DNA (dsDNA) bacteriophages in which membranes are absent [1,5–7,11]. This point is noteworthy in light of recent studies, which implied that DNA packaging machinery in viruses containing an inner membrane layer is fundamentally different from the vertex-portal apparatus of herpesviruses and bacteriophages [12–14]. Specifically, inner membrane–containing viruses were shown to contain putative DNA-packaging ATPases that, in addition to the regular Walker A and B motifs, carry a conserved motif that might act as a membrane anchor [12–14]. The structural aspects that underlie genome translocation mechanisms deployed by these viruses remain, however, largely unknown [15].

The amoeba-infecting virus *Acanthamoeba polyphaga mimivirus* is a member of the nucleocytoplasmic large DNA viruses (NCLDV) clade that comprises several eukaryote-infecting viral families such as the *Phycodnaviridae*, *Iridoviridae*, and *Asfarviridae* [16]*.* As in all members of NCLDVs, the Mimivirus is composed of a core containing a dsDNA genome, which is surrounded by a lipid membrane that underlies an icosahedral capsid [17–19]. The capsid is, in turn, covered by closely packed 120-nm-long fibers that form a dense matrix at their attachment site [17–19]. The closely packed fibers and the dense layer at the base of these fibers represent a unique feature of the Mimivirus. In addition, a single modified vertex has been detected in mature particles [18].

With a 1.2–mega base pair (Mbp) dsDNA genome and a particle size of ∼750 nm, the Mimivirus represents the largest virus documented so far, blurring the established division between viruses and single-cell organisms [17,18,20]. Prompted by these unique features, we conducted high-resolution studies of the Mimivirus life cycle within its amoeba host, focusing on genome delivery and packaging stages that remain poorly understood in all members of the NCLDV clade. By performing cryo-scanning electron microscopy and electron tomography on cryo-preserved host cells at different post-infection time points, we demonstrate that DNA exit occurs in phagosome-enclosed viral particles through a massive opening of five icosahedral faces of the capsid. This large-scale capsid reorganization, which occurs at a unique, structurally modified icosahedral vertex, allows for the fusion of the internal viral membrane with the membrane of the host phagosome. The fusion leads, in turn, to the formation of a massive membrane conduit through which DNA delivery occurs. In conjunction with single-particle reconstruction studies that indicated the presence of two successive membrane layers underlying the Mimivirus protein shell [18], these observations raise the possibility that the Mimivirus genome is released into the host cytoplasm and is translocated toward the host nucleus enclosed within a vesicle that is derived from the viral inner membrane.

We further show that DNA packaging into preformed Mimivirus procapsids proceeds through a non-vertex portal, transiently formed at an icosahedral face distal to the DNA delivery site. Along with comparative genomic studies [12,13], these results imply a viral packaging pathway reminiscent of DNA segregation in bacteria, a pathway that might be common to internal-membrane–containing viruses. Taken together, the observations reported here may indicate that Mimivirus and potentially other large dsDNA viruses have evolved mechanisms that allow them to effectively cope with the exit and entry of particularly large genomes.

## Results

### Massive 5-Fold Assembly on the Mimivirus Capsid

Extracellular Mimivirus particles were sectioned following cryo-fixation and examined by transmission electron microscopy (TEM). Notably, all TEM specimens in the current study were preserved through the high-pressure freezing technique that, in sharp contrast to conventional chemical fixation protocols, allows for instantaneous immobilization of all structures in their native morphology. As such, this preservation method is generally considered to be highly reliable and hence optimal for electron tomography studies [21].

**Figure 1:**
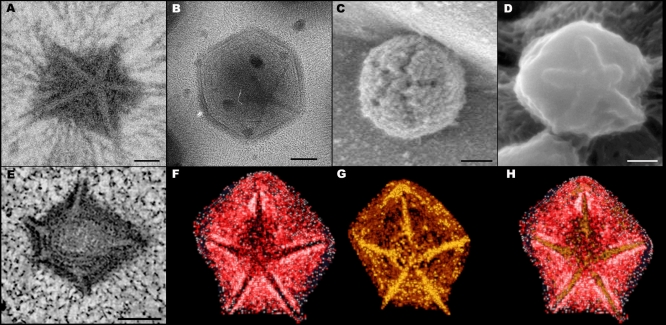
Mimivirus Star-Shaped Structures (A) TEM image of cryo-fixed sectioned and stained extracellular Mimivirus particles revealing a star-shaped structure at a unique vertex. (B) Cryo-TEM image of a whole vitrified fiber-less Mimivirus. (C) SEM image of the star-shaped structure in a mature extracellular Mimivirus particle. (D) Cryo-SEM of an immature, fiber-less particle. (E) Tomographic slice of a mature intracellular Mimivirus particle captured at a late (12 h post infection) infection stage. As shown in Video S1, at this late stage the host cell is packed with mature viral particles. (F and G) Volume reconstruction of the particle shown in (E), revealing the presence of an outer (red) and inner (orange) capsid shells. The star-shaped structure is present in both shells but adopts partially open (dark, star-like region), and completely sealed configurations in the outer and inner shells, respectively. (H) Superposition of the two shells in (F) and (G). Scale bars, 100 nm in (A, B, D, and E), and 200 nm in (C).

The extracellular particles reveal an unprecedented 5-fold star-shaped structure that is localized at a single icosahedral vertex and extends along the whole length of the five icosahedral edges that are centered around this unique vertex (Figure 1A). Geometric considerations of an icosahedron structure modified along five icosahedral edges that is randomly sliced indicate that if all viral particles include such a massive assembly, parts of this structure should be discerned in 75%–80% of the sections used for TEM analysis, depending on the thickness (70–80 nm) of the sections. In ∼500 extracellular viruses examined, the 5-fold assembly or parts thereof were detected in ∼400 particles (80%), thus demonstrating that all viral particles contain this structure. None of the examined extracellular viral particles or of the intracellular particles (see below) revealed more than one star-shaped structure per particle, a finding fully consistent with single-particle cryo-TEM studies in which a single modified vertex was detected [18].

The presence of the star-shaped assembly was further confirmed by cryo-TEM studies conducted on whole extracellular Mimivirus particles that were vitrified in their hydrated state. Due to the interference of the extremely dense fiber layer that surrounds the viral capsids [18], the 5-fold structure could not be detected in mature particles, but was clearly and consistently discerned in immature, fiber-less viruses that constitute a small yet significant (∼10%) population of the viruses that are released upon lysis of the amoeba cells at the completion of the infection cycle (Figure 1B).

To ascertain that the 5-fold assembly represents a general and genuine feature, >500 extracellular Mimivirus particles were analyzed by cryo-scanning electron microscopy (cryo-SEM). These studies corroborate the presence of a massive 5-fold structure at a unique vertex of the particle. The assembly is detected in fiber-covered Mimivirus where it appears as crevices, but is particularly conspicuous and consistently revealed in immature fiber-less particles, where it takes the form of prominent ridges (Figure 1C and 1D, respectively). The crevices that characterize the 5-fold structure in mature particles (Figure 1C) imply that this particular structure is depleted of fibers, in contrast to all other regions of the capsid.

Electron tomography (Figure 1E and Video S1) and volume-reconstruction analyses (Figure 1F–1H) were performed on viral particles within infected amoeba cells at final infection stages (12 hours post-infection), where cells are crammed with mature viruses. The analyses indicate that the Mimivirus capsid is composed of two superimposed shells characterized by conspicuously different densities. This observation, obtained from three tomography analyses conducted on different intracellular viral particles, is consistent with single-particle reconstruction studies, which indicated the presence of a protein shell surrounded by a distinct layer that corresponds to a dense base of fibers [18]. In addition to the two shells, a prominent star-shaped structure is discerned in the intracellular Mimivirus particles (Figure 1E). Volume-reconstruction analysis of the star-shaped structure indicates that the outer shell adopts a partially open configuration (corresponding to the dark star-shaped region in Figure 1F). This open region is, however, completely sealed by the underlying inner shell (Figure 1G), an observation compatible with the ridges that delineate the 5-fold star-shaped structure in immature fiber-less particles (Figure 1D). Figure 1H, which represents a superposition of the two shells, demonstrates the perfect match between the ridges at the inner shell and the regions in which the outer shell is missing.

### The Stargate: A 5-Fold Structure That Acts as a Genome Delivery Portal

**Figure 2:**
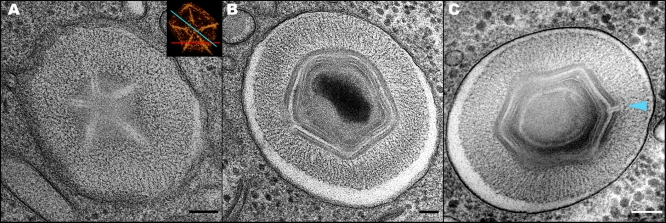
Morphological Aspects of Phagosome-Enclosed Mimivirus Particles (A) TEM projection of a phagosome-enclosed particle sectioned along a plane that contains the whole star-shaped structure. The observed features are similar to those characterizing extracellular Mimivirus particles (Figure 1A) and mature intracellular particles (Figure 1E), thus indicating that the star-shape assembly is present in the capsid throughout the life cycle of the Mimivirus. The inset provides the various possibilities for random sectioning of the Mimivirus particle. (B) TEM projection of a phagosome-enclosed particle sectioned along a plane that does not contain the star-shaped assembly, thus revealing only unmodified vertices. (C) TEM projection of a phagosome-enclosed Mimivirus particle, revealing the star-shape structure (blue arrowhead) sliced in its center along the plane depicted by a blue line in the inset in (A). Scale bars, 100nm.

**Figure 3:**
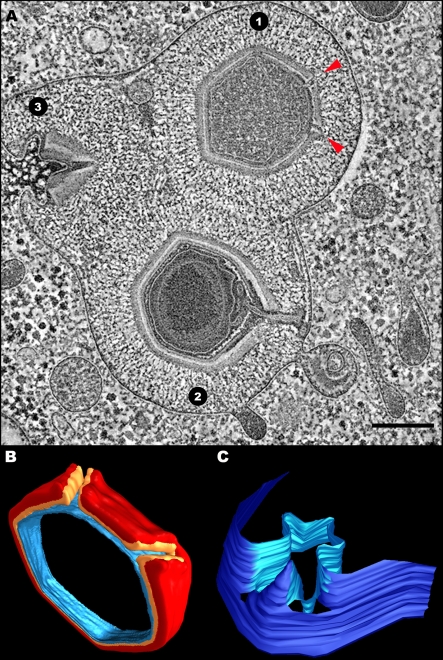
Mimivirus Uncoating and Membrane Fusion (A) Tomographic slice of a late phagosome enclosing three Mimivirus particles at early, advanced, and final uncoating stages (particles 1, 2, and 3, respectively). At the early uncoating stage, a partial opening of the inner protein shell at the stargate assembly is initiated. The red arrowheads highlight the star-shaped structure sectioned along the plane depicted by a red line in the inset in Figure 2A. The opening of the stargate allows for the extrusion of the viral membrane towards the phagosome membrane, a stage characterizing particle 2. In the final uncoating stage, fusion between viral and phagosome membranes occurs, as revealed in particle 3. The lysosomes surrounding the phagosome should be noted. The reconstructed volume of the tomographic slice is shown in Video S2. Scale bar, 100nm. (B) Volume reconstruction of particle 1 in (A), showing the outer (red) and inner (orange) capsid shells and the closely apposed inner membrane (light blue). The opening of the star-shaped structure in the inner shell (in contrast to its closed configuration in extracellular particles or in particles enclosed in early phagosomes) should be noted. (C) Surface rendering of particle 3 in (A), showing fusion of the viral and phagosome membranes (light and dark blue, respectively) at the site of the opened star-shaped structure. The boundary between the viral and phagosome membranes is arbitrary.

A recent study implied that the initial stages of Mimivirus infection occurs by phagocytosis [19]. Our observations support this notion by demonstrating the presence of phagosomes containing one or several viral particles within infected amoeba cells at early (2–3 h) post-infection time points (Figures 2 and 3).

The different morphological aspects revealed by the phagosome-enclosed viral particles are straightforwardly interpreted as a result of sectioning the viruses along different planes, as clarified in the inset in Figure 2A and demonstrated in Video S2. Specifically, in a randomly sliced section that contains the star-shape assembly and is parallel to field of view depicted in the inset, a star-shape structure is detected, as demonstrated in Figure 1A, 1E–1H, and in Figure 2A. If, on the other hand, the TEM section is sliced along a plane parallel to that illustrated in the inset yet located below the star-shape assembly, only unmodified vertices will be detected, as indeed is the case for the viral particle shown in Figure 2B. Sections perpendicular to a single star-like assembly should reveal either one or two modified vertices, which correspond to slices along the blue and red lines in the inset, respectively. These two morphological aspects are indeed manifested by the viral particles shown in Figure 2C and in Figure 3A (particle 2), which reveal a single modified vertex, and by particle 1 in Figure 3A, in which two modified vertices (marked by red arrowheads) are visible. Such two modified icosahedral edges that belong to the same star-shaped assembly are particularly evident in thick sections that are sliced along the red line in the inset, as indeed shown in Figure 3B and in Video S2. In conjunction with the geometric considerations described above, a statistical analysis conducted on more than 100 phagosome-enclosed viral particles (which basically represent mature virions) indicate that all intracellular Mimivirus particles contain a modified, star-shaped vertex, and that this vertex is unique, as is the case for the extracellular Mimivirus particles.

Figure 3A shows a tomographic slice of a phagosome in which three viral particles were captured at three successive uncoating stages. Volume reconstruction of the particle 1 (early uncoating) reveals that in this virus, both the outer (red) and inner (orange) capsid layers are opened at the star-shaped assembly (Figure 3B). The opening of both shells is in contrast with the morphology revealed by extracellular viruses (Figure 1), as well as by intracellular particles during early phagocytic stages (Figure 2C), in which the inner shell appears to be completely sealed. This opening allows for the lipid layer underlying the capsid shell (blue layer in Figure 3B) to protrude and extend towards the phagocytic membrane. This stage is represented by the viral particle 2 in Figure 3A. The final uncoating stage is demonstrated by the particle 3 (Figure 3A). Surface rendering analysis of this virus demonstrates a massive opening of five triangular icosahedral faces that occurs at the star-shaped vertex and results in a fusion of the viral (light blue) and phagocytic (dark blue) membranes (Figure 3C). The three uncoating stages are visible in the tomogram shown in Video S2.

**Figure 4:**
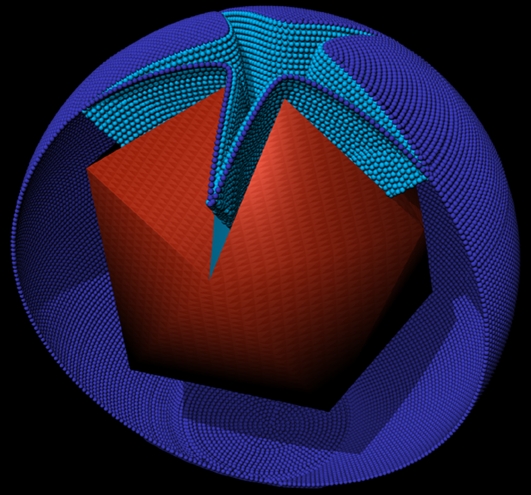
Schematic Representation of a Mimivirus Particle at Its Final Uncoating Stage The capsid (red) is opened at the stargate, allowing for fusion of the viral and phagosome membranes (light and dark blue, respectively), thus forming a star-shaped membrane conduit (See Video S2 for the tomogram from which the model was derived).

We interpret these observations as indicating that the star-shaped structure, which we coin “stargate”, represents a device that mediates a large-scale capsid opening, thus allowing for the protrusion of the inner viral membrane and a subsequent viral-phagosome membrane fusion. This fusion results in the formation of a massive membrane tube through which the genome core is released into the host cytoplasm. The notion that DNA delivery occurs following the formation of a membrane conduit is supported by the presence of empty capsids within phagosomes (our observations and [19]). The large-scale capsid opening at the stargate site, and the membrane tube are depicted in a schematic model (Figure 4), which is based on the tomography (Figure 3A and Video S2) and surface rendering (Figure 3C) of particle 3 in Figure 3A.

**Figure 5:**
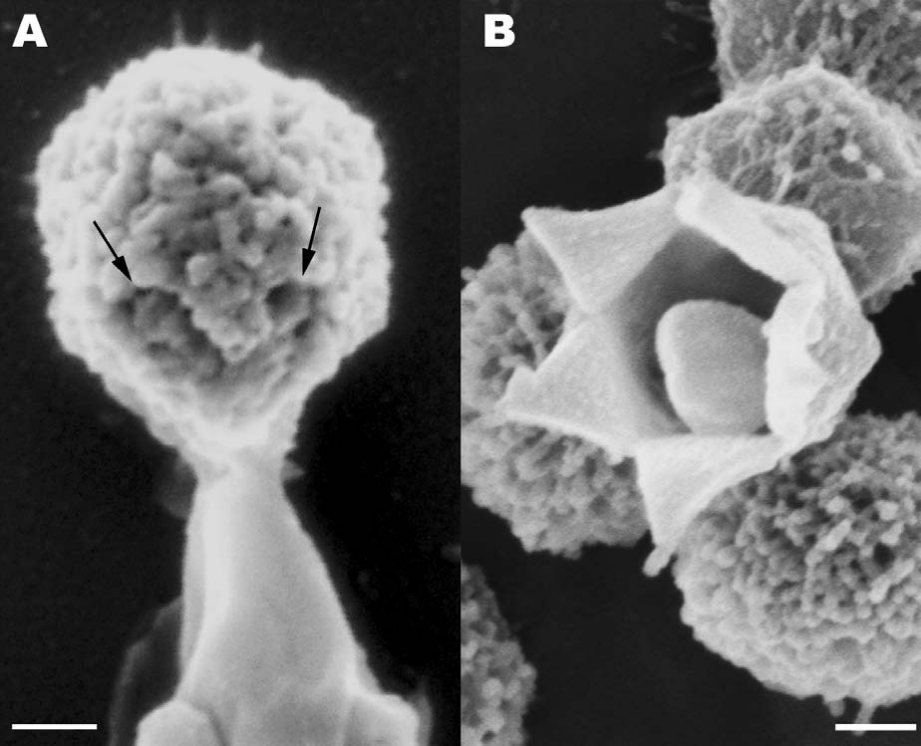
**** Stargate Opening (A) SEM image depicting the release of membranal structures following exposure of mature, extracellular Mimivirus particles to 83 °C for 30 min. Membrane release consistently occurs at the stargate (arrows pointing to the fiber-less edges of the stargate). (B) Extracellular Mimivirus particle revealing a conspicuous 5-fold opening. Such open stargates were detected in a small population of extracellular particles, and may represent empty viral particles released upon the viral-induced lysis of the host cells. Scale bars, 100nm.

To identify the factors that promote the stargate opening within the host phagosome, and in light of extensive fusion of lysosomes with phagosomes in which viral uncoating occurs (Figure 3A and Video S2), isolated Mimivirus particles were exposed to acidic conditions (pH 6.5, 5.5, and 4.5) in the absence or presence of lysozyme. None of these treatments triggered stargate opening, implying that other or additional factors are involved in effecting this structural reorganization. Exposure of particles to elevated temperature (83 ^°^C) for 30 min resulted in a release of membranal structures that specifically occurred at the stargate site in ∼10% of the particles (Figure 5A). While physiologically irrelevant, this finding implies that the stargate represents a structurally susceptible site, a conjecture further supported by the observation that a small population (<1%) of extracellular particles reveals a conspicuous 5-fold opening (Figure 5B). These capsids might represent faulty viral particles, or particles that have ejected their genome and then released to the medium upon viral-induced lysis of the host cells at the completion of the infection cycle.

### Viral Factories and DNA Packaging

**Figure 6:**
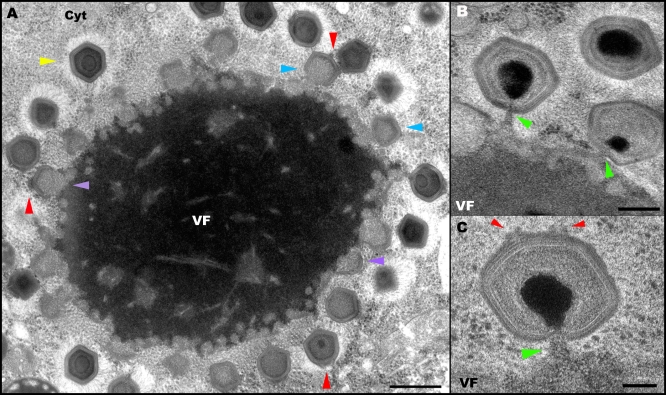
Intracellular Viral Factories and DNA Packaging (A) TEM of an intracellular viral factory, revealing Mimivirus particles at various assembly stages. Empty, fiber-less particles at initial assembly stages, appearing at close vicinity to the periphery of the viral factory (purple arrowheads); partially assembled empty, fiber-less particles (blue); and mature, fiber-covered particles located further away from the viral factory than immature particles (yellow). A stargate that is consistently located at the distal site of the factory can be discerned in several particles (red arrowheads). VF and Cyt stand for viral factory and cytoplasm. (B and C) TEM of Mimivirus particles undergoing DNA packaging through a non-vertex face-centered site (green arrowheads). Two edges of a stargate located at the opposite site of the DNA packaging site are indicated with red arrowheads. Approximately 60% of viral particles undergoing DNA packaging (∼120 particles out of ∼200 analyzed in this study) reveal a face-centered DNA packaging site, while in other virions the packaging site is unclear. As discussed in the text, the reason for this apparent ambiguity in the packaging site results from intrinsic constraints in the interpretation of data derived from projection TEM studies of thin sections. More inclusive data on this issue can, however, be obtained from electron tomography analyses of thick sections, as demonstrated in Figure 7 and in Video S3. Scale bars: 500 nm in (A), 200 nm in (B), 100 nm in (C).

**Figure 7:**
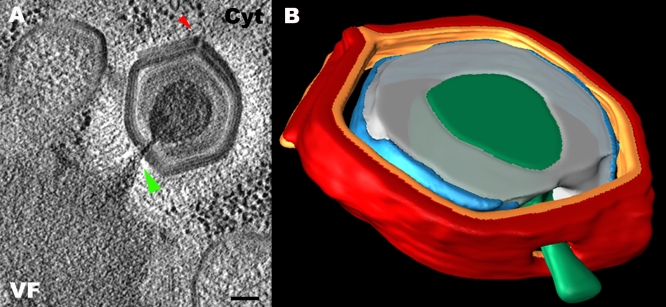
**** Electron Tomography of a DNA Packaging Site (A) Tomographic slice of a procapsid undergoing DNA packaging. The DNA-loaded packaging gateway and the stargate are highlighted (green and red arrowheads, respectively). Scale bar, 100 nm. (B) Volume reconstruction of the particle depicted in (A) showing the orifice through which DNA (green) is packaged, which spans the outer and inner capsid shells, and the internal membrane (red, orange, and blue, respectively). The protein core underlying the membrane is shown in gray (See Video S3 for the whole tomogram).

Following release, the Mimivirus genome is imported into the host nucleus and then translocated to a cytoplasmic viral factory where viral assembly occurs [19]. TEM studies of infected and cryo-fixed amoeba cells reveal that already at 8 h post-infection, viral factories are studded with empty, fiber-less procapsids that are only partially assembled, as well as with icosahedral procapsids undergoing DNA packaging (Figures 6 and 7) [19]. The occurrence of DNA packaging into procapsids at the periphery of the factories (green arrowheads in Figures 6 and 7) was supported by specific DNA staining and Br-dU experiments (unpublished data). Intriguingly, in some particles, the genome appeared to be translocated at a vertex (Figure 6A) [19], whereas in others, DNA translocation proceeds through an aperture located at an icosahedral face (Figure 6B and 6C). A statistical survey of a large number (>50) of intracellular viral factories indicated that at any thin section of the factory analyzed in TEM, 20–25 viral particles are present at various stages of assembly. Out of these assembling virions, 4–5 particles were captured at the stage of DNA packaging, and within this population, packaging through a face-located aperture, as shown in Figure 6B and 6C, was consistently detected in 2–3 virions. Thus, in more than 200 analyzed particles that undergo DNA packaging, a face-centered rather than a vertex-centered packaging is visible in more than 120 (∼60%) particles.

Projection images derived from TEM studies of thin sections cannot provide, however, unequivocal data on the precise site of the packaging process, as such data can be masked or incorrectly interpreted due to the angle of the site within the TEM section relative to the electron beam. To obtain deeper insights into the DNA packaging process in Mimivirus, we performed electron tomography and volume reconstruction analyses on three randomly chosen procapsids during their assembly on the periphery of the viral factories. A slice of a tomogram obtained from one of these assembling procapsids (Figure 7A; the whole tomogram is shown in Video S3) demonstrates that DNA packaging proceeds through an aperture that spans the outer and inner capsid shells, as well as the internal membrane, and is located at the center of an icosahedral face. The aperture, which is sealed following completion of DNA packaging as implied by the structure of mature particles, adopts a cone shape with diameters of 35 nm and 20 nm at the outer and inner shells, respectively. These features, clearly discernible in the reconstructed volume of the particle (Figure 7B), are detected in all three tomograms of assembling procapsids. Notably, whenever stargates are discerned in electron microscopy sections of assembling viral particles, they are invariably detected at the distal site of the factory, pointing away from the replication center (Figures 6 and 7). This finding, which is consistent with earlier observations [19], is particularly evident in tomograms obtained from relatively thick sections (Figure 7 and Video S3).

**Figure 8:**
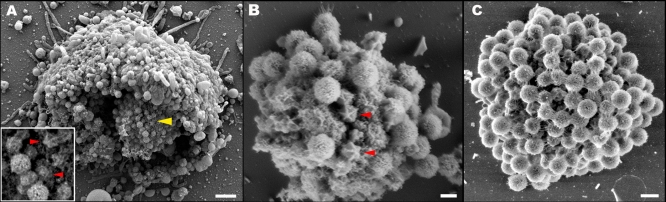
Isolated Viral Factories (A) SEM of a viral factory within an amoeba cell lysed 8 h post infection. A high-magnification image of the site indicated by the yellow arrowhead (inset) shows assembling fiber-less particles with stargates (red arrowheads), as well as mature fiber-coated particles. (B) SEM of a viral factory isolated 8 h post infection. The factory is studded with viral particles at various assembly stages. Stargates are indicated with red arrowheads. (C) SEM of a viral factory isolated 10 hours post infection. Only mature, fiber-covered particles can be detected. Scale bars are 2 μm in (A), 300 nm in (B), and 500 nm in (C).

To substantiate our TEM results, we have isolated viral factories by gentle lysis of infected amoeba cells at 8–10 h post-infection, thus capturing successive assembly stages. SEM studies of factories isolated at 8 h post-infection show immature viral particles that abut on the periphery of the factories and reveal conspicuous stargates (Figure 8A and 8B). Due to the dense fiber layer, stargates are hardly discernible in SEM analysis of mature particles, which are located further away from the periphery. Notably, in viral factories isolated at a 10 h post-infection (Figure 8C), only mature particles, which presumably cover and mask the immature particles, can be detected. Thus, the SEM results, obtained from >50 isolated viral factories, corroborate the TEM studies conducted on intracellular factories, and strongly imply that the stargate structures represent an early stage of the viral assembly.

## Discussion

The observations reported here imply that in contrast to all viral genome translocation processes heretofore characterized, DNA exit and packaging in the amoeba-infecting virus *Acanthamoeba polyphaga mimivirus* proceed through different portals, both revealing unparalleled features.

### The Stargate Assembly

Mimivirus infection is initiated by phagocytosis [19], and genome delivery occurs upon exposure of the virus to cues within the host phagosome. While the nature of these cues remains unknown, detection of multiple lysosomes undergoing fusion with the phagosomes (Figure 3A and Video S2) may imply that lysosomal activity promotes the opening of the viral capsid. The observations reported here indicate that this opening entails a unique portal, the stargate, which is located at a single icosahedral vertex (Figures 1–5), in keeping with previous single-particle studies [18], in which a single modified vertex has been identified. These studies, as well as our electron tomography observations (Figure 1E–1H) revealed that the Mimivirus is composed of a protein shell surrounded by an outer layer corresponding to a dense base of fibers.

Our cryo-TEM (Figure 1B), cryo-SEM (Figure 1C and 1D), and electron tomography of cryo-fixed specimens (Figure 1E–1H) indicate that the stargate is located within the protein shell, extending along the whole length of five icosahedral edges that are centered around a single icosahedral vertex, thus forming an assembly of unprecedented morphology and dimensions. The icosahedral edges appear as prominent ridges in the protein shell, which are clearly discerned in immature, extracellular viral particles that lack the dense fiber layer (Figure 1D). Our observations further indicate that while the outer shell surrounds most of the inner protein shell, it is absent along the icosahedral edges that constitute the stargate (Figure 1F). This fiber-less region is likely to enable the cues that trigger the opening of the stargate to reach their specific target in the inner protein shell. Notably, the stargate is detected in extracellular Mimivirus particles, in phagosome-enclosed virions, in mature intracellular viral particles present within the amoeba host cells at the final infection stage (Figures 1 and 2 and Video S1), as well as in assembling virions (Figures 6–8), thus indicating that this prominent assembly is present in the Mimivirus capsid throughout the virus life cycle.

### Genome Exit: Potential Vesicle-Mediated DNA Release and Translocation

The large-scale conformational change of the capsid whereby the five icosahedral faces centered on the unique stargate vertex open up, allows the extrusion of the viral membrane that underlies the viral protein shell. This extrusion is followed by the fusion of the viral membrane with the phagosome membrane, thus resulting in the formation of a large membrane conduit (Figures 3–5 and Video S2) through which the Mimivirus genome is presumably released into the host cytoplasm. The actual mode of DNA release remains unclear, as in all virus-containing phagosomes inspected in this study (>100), only mature viruses, viruses at various uncoating stages, or empty viral particles could be discerned (Figures 2 and 3).

In light of the size of the Mimivirus genome, the failure to capture genome release is intriguing. On the basis of cryo-TEM studies of Mimivirus particles [18] that implied the presence of two successive membrane layers underlying the protein shell (as is the case for at least one additional member of the NCLDV clade, the African Swine Fever Virus [22,23]), it can be hypothesized that the Mimivirus genome is released into the host cytoplasm enclosed within a vesicle. Such a vesicle might be derived from the inner membrane layer, whereas the outer membrane forms a conduit for this DNA-containing vesicle by fusing with the phagosome. This conjecture provides a rationale to the need for the massive opening of the capsid that is reported here, a possible reason for the failure to capture DNA release (as a vesicle-mediated release would likely be a fast process), as well as a plausible answer to the question how is the viral genome protected against host nucleases during its transport to the host nucleus. Moreover, the notion of a vesicle-mediated exit and transport of the Mimivirus genome provides a potential and highly attractive solution to the question of how is a 1.2-Mbp DNA molecule translocated through the extremely crowded cytoplasm of the host, which has been shown to present a supreme barrier for translocation of long DNA molecules [24]. The notion of genome release and transportation within a vesicle that is derived from internal viral membranes is, to the best of our knowledge, unprecedented and is being currently investigated.

Notably, while DNA injection and packaging in the internal-membrane–containing tail-less bacteriophage PRD1 appear to occur through a unique vertex [25], in vitro studies implied that PRD1 delivers its genome through a membrane tube [26]. For this to occur, the PRD1 capsid must open up in a yet uncharacterized process that might be similar to the genome release process occurring in Mimivirus. High-resolution structural studies of PRD1 life cycle will be required to address this intriguing possibility.

Mimivirus assembly occurs in cytoplasmic viral factories [19]. DNA is packaged into preformed procapsids located at the periphery of factories (Figure 6)[19]. Studies of intracellular factories (Figures 6 and 7) as well as of viral factories isolated at various post infection time points (Figure 8) indicate that the formation of the stargate structure occurs at a very early stage of the viral assembly. These observations imply that in addition to acting as a DNA release portal, the stargate might be involved in the initiation of Mimivirus particles assembly. Such an initiation role is in keeping with the fact that capsids incorporate only one portal that is located at a unique vertex, and this symmetry-breaking step can only be rationalized in terms of a singular event, as is the initiation stage. Indeed, previous studies indicated that portals are involved in the initiation of capsid assembly in herpesviruses and several bacteriophages such as T4 and SPP1 [27,28]

### Genome Packaging through a Face-Located Portal

Our electron tomography and volume reconstruction analyses, supported by TEM and SEM studies, demonstrate that DNA packaging in Mimivirus proceeds through a transient aperture located at a distal site of the stargate site. These studies further indicate that in contrast to all heretofore-characterized viruses, Mimivirus genome packaging occurs at an icosahedral face rather than at a vertex (Figures 4–6 and Video S3). Notably, 3-nm-wide pores detected on the 3-fold axes in “open” procapsids of the α3 bacteriophage of the *Microviridae* family were proposed as possible DNA entry sites [29,30]. In the current study, such a face-centered, non-vertex, DNA packaging site is directly demonstrated. The functional significance of this finding becomes apparent in light of recent biochemical and comparative genomic studies, which indicated that inner-membrane–containing viruses such as bacteriophage PRD1 and members of the NCLDV clade (including Mimivirus) code for proteins that are closely homologous to ATPases of the FtsK/SpoIIIE/HerA superfamily [12–14]. These ATPases were proposed to act as membrane-anchored motors that pump DNA through a closing membranal septum during bacterial and archaean division [31,32]. On the basis of these findings and considerations, it has been suggested that viruses containing inner membranes package their genomes through a pumping mechanism akin to the DNA segregation pathway deployed in bacteria and archaea [12–14].

Our observations complement this conjecture. Since FtsK/SpoIIIE/HerA ATPases mediate a strictly unidirectional mode of DNA translocation [31,32], this system is unlikely to be responsible for both exit and packaging of viral genomes. In keeping with this notion, we identify distinct exit and entry portals in Mimivirus. Moreover, whereas a vertex-centered motor for DNA packaging in bacteriophages and herpesviruses represents a thermodynamically sensible solution, because it minimizes vertex-portal interactions, such a setting would be incompatible with a pumping system that must rely on robust motor–membrane interactions. Such interactions can, however, be maximized when the packaging motor is located within an icosahedral face (rather than on an icosahedral vertex). In addition, our conjecture that the DNA entry portal is sealed once packaging is concluded is consistent with recent studies that indicated that the DNA translocating ATPase SpoIIIE promotes membrane fusion following completion of bacterial DNA segregation [33]. Interestingly, the cone-shaped aperture through which DNA is packaged is characterized by a diameter of 20 nm at the inner shell, thus capable of accommodating more than a single DNA duplex, as indeed is implied by TEM studies (Figures 6 and 7). Of note in this context is the conjecture that several SpoIIIE rings might fuse to form a larger ATPase ring [31].

### Evolutionary Considerations

The size and genome complexity of the Mimivirus call into question the conventional division between viruses and single-cell organisms. Our findings, which support the conjecture that the DNA packaging mechanism deployed by internal-membrane–containing viruses might share structural and functional patterns with bacterial DNA segregation [12–14], further substantiate the notion that the conventional division between viruses and single-cell organisms should be re-examined. Moreover, the observations concerning the stargate and its massive opening, the DNA packaging machinery, as well as the possibility raised here that the exit and transportation of the genome occur within a vesicle derived from a viral internal membrane, may indicate that Mimivirus and potentially other large dsDNA viruses have evolved mechanisms that allow them to effectively cope with the exit and entry of particularly large genomes.

Because structure, rather than genomic sequence, represents the most reliable determinant for viral lineage [34], the structural features underlying the Mimivirus replication cycle raise intriguing questions. The presence of distinct portals for genome exit and entry, as well as the shape of the stargate and the unprecedented face-centered location of the packaging portal, may indicate that Mimivirus represents a unique specimen. It is, however, enticing to suggest that these features, along with their functional and evolutionary implications, are shared by diverse viruses containing internal membranes. This conjecture, which is consistent with comparative genomic studies [12,13,16,34], as well as with the notion that an inner membrane represents a key factor for viral evolution and classification [35,36], is being currently tested by high-resolution studies of the replication cycles of various inner membrane-containing viruses. Finally, for only a small fraction of the open reading frames in Mimivirus, genome function has been attributed [17]. The observations reported here may stimulate further studies on the Mimivirus that will focus on heretofore uncharacterized structural features, including the stargate, its putative role in Mimivirus assembly, and its massive opening, as well as the face-centered DNA packaging apparatus. Such studies are likely to provide deeper insights into the unusually complex genome of this virus and into the factors that directed and dictated its evolution.

## Material and Methods

### Sample preparation for TEM and electron tomography.

Acanthamoeba polyphaga were cultivated and infected by Mimivirus as previously described [20]. Infected cells at various post infection time points were cryo-immobilized by the high-pressure freezing technique [21], using an HPM high-pressure freezer (BAL-TEC). Samples were then freeze-substituted (Leica EM AFS) in dry acetone containing 2% glutaraldehyde and 0.1% tannic acid for 60 h at −90 °C, and warmed up to room temperature over 24 h. Following acetone rinses, samples were incubated in 0.1% uranyl acetate (UA) and 1% OsO_4_ for 1 h, infiltrated with increasing concentrations of Epon over 6 d, and polymerized at 60 °C. Thin sections (50–70 nm), obtained with an Ultracut UCT microtome (Leica) were post-stained with 1%–2% uranyl acetate and Reynold's lead citrate and examined using FEI Tecnai T12 TEM operating at 120 kV. Images were recorded on a MegaView III CCD (SIS). Preparation of vitrified Mimivirus and cryo-TEM studies were as described in [18].

For electron tomography, semi-thick sections (170–200 nm) decorated on both sides with 12-nm colloidal gold markers were prepared as described above, and post-stained with 2% UA. Double-tilted image series were acquired in FEI Tecnai F-20 TEM operating at 200 kV. Images were recorded on a 4kx4k TemCam CCD camera. Acquisition was performed at 1° intervals over a range of ±68°, using SerialEM program [37]. Alignment and 3D reconstruction were performed with IMOD image-processing package [38]. IMOD and Amira 4.1 packages were used for modeling.

### Sample preparation for SEM and cryo-SEM.

Viruses purified by filtration were fixed with 2% gluteraldehyde in Cacodylate buffer for 1 h. Viruses were deposited on poly-L-lysine–treated formvar-coated 200-mesh Ni grids, post-fixed with 1% OsO_4_, 1% tannic acid, and 1% uranyl acetate. Dehydration in increasing ethanol concentrations was followed by critical point drying using CPD30 (BAL-TEC). Samples were sputter-coated with 2-nm Cr and visualized in the high-resolution SEM FEG Ultra 55 (Zeiss). For cryo-SEM experiments, samples were fixed in 2% gluteraldehyde for 1 h, washed with DDW and deposited on Aclar disk (EMS). Samples were frozen by plunging in liquid ethane, freeze-dried for 1 h at −100 °C in a BAF60 freeze-fracture device (BAL-TEC) and rotary shadowed at 45° with 2-nm platinum-carbon and 5-nm carbon at −120 °C. Samples were transferred to Ultra 55 SEM using a VCT100 vacuum-cryo-transfer, and observed at −120^ o^C.

Replication factories were isolated using the spheroplast methodology [39,40]. Specifically, Acanthamoeba polyphaga were cultivated and infected by Mimivirus. The infected cells were washed with ice-cold 20 mM potassium phosphate buffer, pH 6.5, at different times post infection, and transferred into a glass tube at a concentration of 10^5^ cells/ml. The cells were diluted with two volumes of ice-cold DDW, and incubated for 10 min on ice. The swollen cells were then incubated in ice-cold 0.3 M NaCl in 20 mM potassium phosphate buffer, pH 6.5 for 10 min. Aliquots of 10 μl were deposited on top of 40 μl fixative 1 (4% paraformaldehyde in 20 mM potassium phosphate, 0.2 M sucrose, pH 6.5) and spun onto poly-L-lysine-treated silicon chips at 4000*g* in a swing out rotor for 5 min. The samples were further fixed in fixative 2 (2% glutaraldehyde, 0.2% tannic acid in 20 mM potassium phosphate, 0.2 M sucrose, pH 6.5) for 10 min. The chips were washed in DDW and treated with 1% OsO_4_ (DDW) for 10 min, washed with DDW and stained with 1% UA (DDW) for 10 min. Samples were then prepared for SEM analysis by ethanol dehydration followed by critical point drying. The dried samples were coated with 2-nm chromium in a stage-rotation mode. The 5 × 5 mm silicon chips were pre-treated with 0.1% poly-L-lysine (in DDW) and incubated in a humid chamber over-night at 4 °C.

## Supporting Information

Video S1 Electron Tomogram of an Infected Amoeba Host Cell at Late Infection Stage The tomogram includes a large number of mature, intracellular Mimivirus particles at a late (12 h post infection) stage of the infection cycle. At this stage, the virions are crammed within the host cytoplasm. All morphological aspects of the stargates can be discerned in the various viral particles, as function of the sectioning plane. Each frame of the movie is an average of ten 0.9-nm slices of a thick section of the amoeba host cell.
(6.50 MB MPG)

Video S2 Electron Tomogram of a Late Phagosome Enclosing Three Mimivirus Particles at Early, Advanced, and Final Uncoating Stages (Figure 3A) In the particle at the upper right side of the phagosome, the vertex containing the stargate is clearly visible. The tomogram provides an interpretation to the observation that two vertices appear to be modified, by showing that these two sites actually belong to the same vertex and correspond to the stargate edges. The extrusion of the viral membrane towards the phagosome membrane is visible in the particle at the bottom on the right side of the phagosome. The opening of the stargate and formation of the membrane tube through fusion of the viral and phagosomal membranes are indicated in the particle at the left region of the phagosome. Note the fusion of lysosomes with the phagosome. Each frame of the movie represents an average of ten 0.9-nm slices of a thick section of a Mimivirus particle.
(4.05 MB MPG)

Video S3 Electron Tomogram of a Procapsid Undergoing DNA Packaging at the Periphery of a Viral Factory (Figure 7A) Note the stargate structure at the opposite site of the DNA packaging site. Each frame of the movie is an average of ten 2.7-nm slices of a thick section of a Mimivirus particle.
(2.02 MB MPG)

## Abbreviations

cryo-SEM: cryogenic scanning electron microscopy
dsDNA: double-stranded DNA
NCLDV: nucleocytoplasmic large DNA virus
TEM: transmission electron microscopy
